# Health-Promoting Phytochemicals from 11 Mustard Cultivars at Baby Leaf and Mature Stages

**DOI:** 10.3390/molecules22101749

**Published:** 2017-10-17

**Authors:** Marissa D. Frazie, Moo Jung Kim, Kang-Mo Ku

**Affiliations:** 1 Division of Animal and Nutritional Sciences, West Virginia University, Morgantown, WV 26506, USA; mdfrazie@mix.wvu.edu; 2 Division of Plant and Soil Sciences, West Virginia University, Morgantown, WV 26506, USA; moojung.kim09@gmail.com

**Keywords:** mustard, phytochemicals, glucosinolates, isothiocyantates, carotenoids, phenolic, anthocyanin, antioxidant

## Abstract

Mustard is a *Brassica* vegetable that provides a number of phytonutrients. However, the phytonutrient profile of mustard has been relatively limited. We analyzed the glucosinolates and their hydrolysis products, carotenoids, total anthocyanin and phenolic contents, and antioxidant capacity of the leaves of 11 mustard cultivars grown in a greenhouse at the baby leaf and mature stages. An aliphatic glucosinolate sinigrin and its hydrolysis products allyl isothiocyanate and 1-cyano-2,3-epithiopropane were the major phytonutrients in the mustard leaves. Carotenoids β-carotene, lutein, violaxanthin, and neoxanthin were detected. We found phytonutrient concentration and their change with plant growth were cultivar-dependent. The %RDA value for vitamin A calculated using β-carotene content and retinol activity equivalents suggests that mustard cultivars used in this study can be a good source of vitamin A. Phenolic contents and antioxidant capacity also varied among cultivars and between physiological stages. Our results suggest that mustard leaves are rich in various phytochemicals and their composition depends on cultivar and the physiological stage. This is the first report on phytochemical composition in various mustard cultivars at different physiological stages.

## 1. Introduction

Mustard (*Brassica juncea* L.) is a *Brassica* vegetable that contains various health-promoting phytochemicals including carotenoids, phenolic compounds, and glucosinolates. These compounds are often associated with their ability to act as detoxifiers against oxidative stress [1]. This is of particular interest for human health, considering that an imbalance of oxidants and antioxidants in the body can lead to the development of certain chronic diseases, such as cancer, diabetes, and cardiovascular disease [2].

Various antioxidants, including phenolic compounds that possess potential health benefits against chronic disease development [3], are found in mustard leaves. Phenolic compounds greatly contribute to the antioxidant capacity of vegetables, partially explaining why there are various antioxidant assays to estimate the antioxidant capacity of vegetables and other foods. Phenolic compounds have also been analyzed for their ability to neutralize free radicals [4]. In addition, phenolic compounds and phenolic-containing leafy vegetables have shown potential health benefits. For instance, Chu et al. [2] studied 10 vegetables and reported that vegetables containing over 50 mg gallic acid eqiv./100 g of total phenolics demonstrated antiproliferative activity on human liver cells HepG2, while vegetables with lower phenolic content showed relatively lower antiproliferative activity. Likewise, anthocyanins, which are water-soluble red-purple pigments that are found in the leaf tissue of certain mustard cultivars, act as antioxidants [5].

Mustard leaves possess antioxidant properties and other potential health benefits from carotenoids as well. Among various carotenoids in mustard leaves, β-carotene is especially of interest for human health, as it possesses provitamin A activity and anticarcinogenic effects [6]. Vitamin A is an essential nutrient that includes retinoids, which also aids in immunity, reproduction, vision, and cell development [7]. In impoverished countries, it is estimated that vitamin A deficiency affects 125–130 million pre-school age children and 7 million pregnant women [8]. Also, vitamin A deficiency contributes to approximately 650,000 premature childhood deaths annually [9]. Other carotenoids such as lutein have also been studied for their role in human nutrition and health [10].

Glucosinolates are another type of phytochemical present in *Brassica* crops and sinigrin, glucobarbarin, and gluconasturtiin have been detected as the major glucosinolates in mustard [11]. Glucosinolates produce various hydrolysis products by an endogenous enzyme myrosinase as a defense mechanism in response to plant tissue damage [12]. It has been reported that the major hydrolysis products of mustard leaves included allyl isothiocyanates (AITC) from sinigrin [13,14]. AITC is responsible for the pungency of mustard, but it are also associated with chemopreventive properties, due to its involvement in phase II detoxification enzymes including glutathione-S-transferase and quinone reductase [15]. In addition to myrosinase, epithiospecifier protein (ESP) greatly affects the formation of glucosinolate hydrolysis products by inhibiting the formation of isothiocyanates and inadvertently increasing epithionitrile formation [16]. For instance, sinigrin mainly produces 1-cyano-2,3-epithiopropane (CETP) rather than AITC under high ESP activity. Therefore, understanding not only glucosinolate composition but also the formation of hydrolysis products will be important in determining the glucosinolate-derived health-promoting effects and sensorial quality of glucosinolate-containing foods including mustards.

Nonetheless, the nutritional profiles of mustard leaves have been limited in their analysis and quantification across different cultivars at different physiological stages. Immature plants such as microgreens and baby leaf plants have been getting popular, partially due to their intense flavor and tender texture compared to mature plants [17,18]. Considering their growing popularity, immature plants merit research on their nutritional value, which can assist growers in their determination of cultivation regime, as well as consumers in their food choices. We hypothesize that phytochemical concentrations would increase as plants grow and display strong correlations between baby leaf and mature stage plants. Additionally, we also assume that there can be a large variation in phytochemical composition among mustard cultivars. In order to test these hypotheses, we analyzed glucosinolates and their hydrolysis products, carotenoids, and phenolics, as well as antioxidant capacity in 19 day-old (immature, baby leaf stage) and 35 day-old (mature) plants across 11 different mustard cultivars.

## 2. Results and Discussion

### 2.1. Glucosinolates

Glucosinolates are the major phytonutrients in mustard that produce a number of hydrolysis products, including AITC from sinigrin. A total of 11 glucosinolates, glucoiberin, progoitrin, glucoraphanin, gluconapin, 4-hydroxyglucobrassicin, glucoerucin, glucobrassicin, 4-methoxyglucobrassicin, gluconasturtiin, and neoglucobrassicin were detected, with sinigrin being the predominant component of mustard leaves. Sang et al. [19] found a similar glucosinolate profile in mustard leaf tissues, including the major glucosinolates that were detected in this study: sinigrin, 4-methoxyglucobrassicin, glucoraphanin, and neoglucobrassicin. However, quantitative data of glucosinolates in mustard leaf tissue was not provided [19].

**Figure 1:**
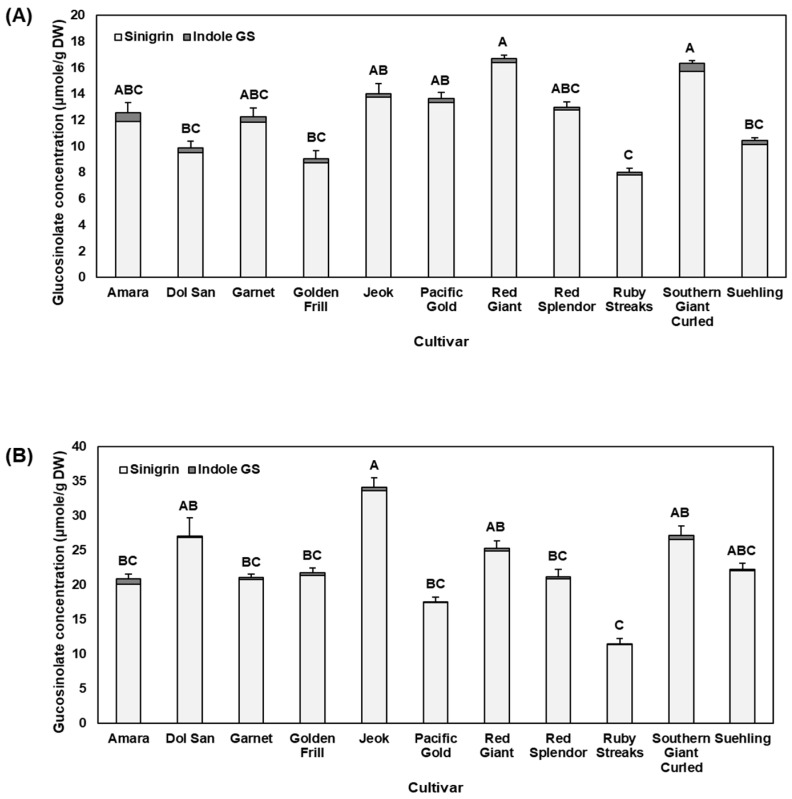
Glucosinolate concentration in the leaves of 11 mustard cultivars at (**A**) baby leaf and (**B**) mature stages. Error bars indicate the standard error of the total glucosinolate concentration (*n* = 3). Different letters indicate significant difference of the total glucosinolate concentration among cultivars at each physiological stage by Tukey’s HSD at *p* ≤ 0.05. GS, glucosinolate.

In baby leaf mustard, sinigrin represented 97.1% of the glucosinolate concentration (Figure 1). Likewise, sinigrin was detected around 98.5% on average in mature mustard leaves. The overall glucosinolate concentration increased from baby leaf to mature mustard. Among investigated cultivars, ‘Ruby Streaks’ contained the lowest concentration of glucosinolates in both baby leaf (8.00 μmole/g DW; Figure 1A) and mature (11.49 μmole/g DW; Figure 1B) mustard. In contrast, ‘Red Giant’ and ‘Jeok’ were the highest in the total glucosinolates in the baby leaf stage (16.74 μmole/g DW) and mature stage (34.16 μmole/g DW), respectively. In general, there were significant correlations for glucobrassicin (*r* = 0.81, *p* = 0.0027, *n* = 11), neoglucobrassicin (*r* = 0.73, *p* = 0.0108, *n* = 11), and total indole glucosinolates (*r* = 0.78, *p* = 0.0048, *n* = 11) between baby leaf stage and mature stage (Table 1). We found a significant difference in the total glucosinolate content among cultivars, and Gupta et al. [20] also reported a large variation of glucosinolate content among 97 lines of mustard.

Differential glucosinolate concentrations at various physiological stages have been reported in a few *Brassica* crops including mustard. Bhandari et al. [21] analyzed glucosinolates of 9 *Brassica* crops from seeds to sprouts and leaves, and glucosinolate concentration in mustard leaves increased from sprout to mature leaves, similar to our result. This suggests that glucosinolates accumulate in the mustard leaves from young, immature stage to mature stage, and therefore mature mustard leaves may provide greater glucosinolate-derived health benefits. Although glucosinolate concentration generally increased from baby leaf to mature plants in the present study, not all the cultivars had the same degree of change. For example, baby leaf ‘Pacific Gold’ (13.66 μmole/g DW) contained the third highest concentration of total glucosinolates, while mature ‘Pacific Gold’ (17.58 μmole/g DW) was ranked at the second lowest concentration of total glucosinolates. This result indicates that accumulation rate of glucosinolates with plant growth differs among mustard cultivars.

### 2.2. Hydrolysis Products and Nitrile Formation (%)

**Figure 2:**
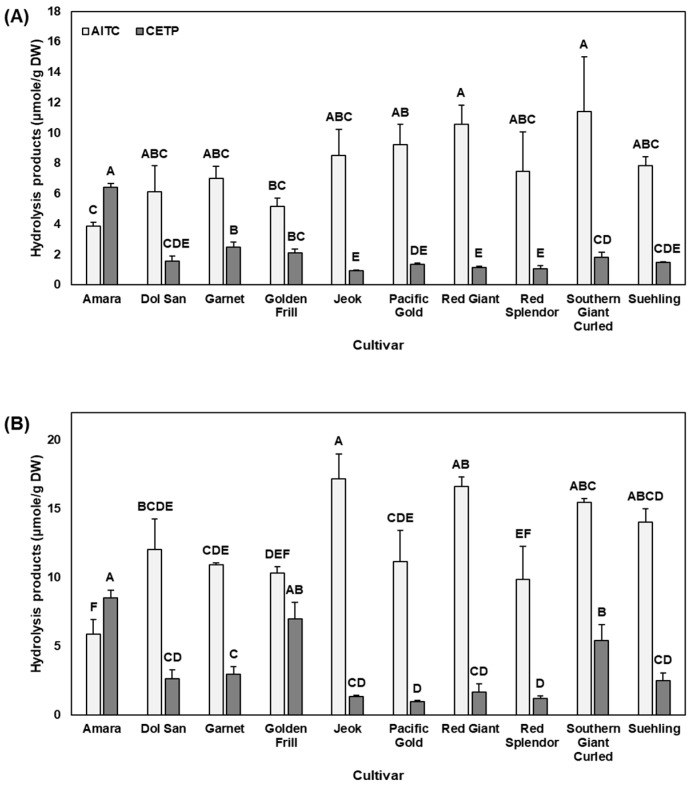
AITC and CETP concentration of 11 mustard leaves at (**A**) baby leaf and (**B**) mature stages. Error bars indicate the standard error of the total hydrolysis product concentration (*n* = 3). Different letters above the error bars indicate significant difference in each hydrolysis product concentration among cultivars at each physiological stage by Tukey’s honest significant difference (HSD) at *p* ≤ 0.05. AITC, allyl isothiocyanate; CETP, 1-cyano-2,3-epithiopropane.

Mustard glucosinolate profiles have been reported, but their hydrolysis products have been less investigated. AITC and CETP were the major hydrolysis products from 11 mustard cultivars investigated in this study. The concentration of AITC and CETP varied within cultivars as well as between physiological stages (Figure 2). In immature mustard leaves, cultivars ‘Red Giant’ and ‘Southern Giant Curled’ were the highest in AITC, while ‘Amara’ mustard was the highest in CETP. In contrast, ‘Jeok’ and ‘Amara’ were the highest in AITC and CETP, respectively, for mature plants. ‘Amara’ was the lowest in AITC in both baby leaf and mature stages.

In general, isothiocyanates are considered as possessing a higher bioactivity than nitriles [22], therefore, enhancing formation of isothiocyanates over nitriles from glucosinolates can result in higher bioactivity. In regulating the formation of isothiocyanates and nitriles, ESP plays an important role, as ESP enhances nitrile formation during hydrolysis of glucosinolates [23]. Therefore, we calculated the relative ratio of AITC, an isothiocyanate, to estimate ESP activity. High nitrile formation was detected in ‘Amara’ in both baby leaf (62.4%) and mature (59.1%) stages (Supplementary Figure S1). In contrast, ‘Jeok’, ‘Pacific Gold’, and ‘Red Giant’ were generally low in nitrile formation (10.1 and 7.4% for ‘Jeok’, 12.9 and 7.9% for ‘Pacific Gold’, and 9.9 and 9.2% for ‘Red Giant’ at baby leaf and mature stages, respectively). Low nitrile formation in these cultivars may indicate a lower ESP activity, which suggests greater health-promoting properties of mustard leaves due to a higher production of isothiocyanates [23]. The relative ratio of AITC to CETP could be a strong indicator of the higher bioactivity of ‘Jeok’, when compared to ‘Amara’ and other cultivars with low glucosinolate concentration in relation to human health benefits. Zhang [24] has reviewed bioavailability and bioactivity of AITC, in particular antimicrobial and anticancer activities. 

Although it is generally accepted that nitriles are not as bioactive as isothiocyanates, how CETP induces gene expression of antioxidant response elements (ARE) was previously reported, supporting their chemopreventive properties [25]. Further studies evaluating bioactivity of various nitriles including CETP will be need to better assess health benefits of mustard and other *Brassica* vegetables. 

AITC has been found to have about a 300-fold stronger odor than CETP [26]. AITC-rich mustard can provide a strong pungent flavor to consumers with chemopreventive effects. Thus, ‘Jeok’, ‘Red Giant’, and ‘Southern Giant Curled’, which have a high level of AITC, can be a good option for those who like pungent flavored-mustard. In contrast, those who like less pungent flavor of mustard would prefer ‘Ruby streaks’ rather than ‘Jeok’, ‘Red Giant’, or ‘Southern Giant Curled’ cultivars. ‘Pacific Gold’ mustard has been widely used as a cover crop, primarily because it is a fast-growing cultivar that controls nematodes and soil fungi. Its AITC content was 4th highest at baby leaf stage and 5th lowest at mature stage among 11 investigated cultivars. However, considering its high yield (Supplementary Table S1), this cultivar can contribute to production of a higher amount of AITC and organic matter. In fact, we found that ‘Pacific Gold’, along with ‘Red Giant’ and ‘Southern Giant Curled’, produced the highest level of AITC per plant at baby leaf stage (Supplementary Figure S2). Similarly, ‘Pacific Gold’ and ‘Red Giant’, as well as ‘Red Splendor’ and ‘Suehling’, produced the highest amount of AITC per plant at mature stage (Supplementary Figure S2). Mustard as a cover crop, or mustard seed meals, have shown their potential benefits for weed control [27,28]. Although some cultivars such as ‘Jeok’ contain high level of AITC, their contribution to control weeds may not be as good as ‘Pacific Gold’ or ‘Red Giant’, partially due to their lower yield compared to these cultivars.

### 2.3. Carotenoids and Recommended Dietary Allowances (RDA)

Neoxanthin, violaxanthin, lutein, and β-carotene were detected in all mustard cultivars investigated in this study (Table 2). These carotenoids have been reported in other *Brassica* vegetables as well, such as kale [29,30]. Additionally, Lefsrud et al. [30] reported a higher level of β-carotene and lutein in 3–4 weeks old leaves than in younger leaves of ‘Winterbor’ kale. They suggested that leaves older than 4 weeks decreased in theses carotenoids, possibly related to senescence. However, we observed that the change in carotenoids depended on compound as well as cultivar. For instance, β-carotene in ‘Jeok’ did not differ between baby leaf and mature stages, but violaxanthin in mature ‘Jeok’ was higher than in baby leaf stage (Table 2). In contrast to increased violaxanthin in ‘Jeok’, violaxanthin concentration in most other cultivars was not different between baby leaf and mature stages. ‘Jeok’ at both physiological stages (1438 and 1386 μmole/g DW at baby leaf and mature stages, respectively) had the highest concentrations of β-carotene, while ‘Amara’ (476.2 μmole/g DW) was the lowest in β-carotene among plants at baby leaf stage. ‘Amara’, ‘Southern Giant Curled’, and ‘Suehling’ were the lowest in β-carotene for mature plants (Table 2). The lutein and β-carotene concentrations found in this study were similar or lower than the results that de Azevedo and Rodriguez-Amaya [29] and Lefsrud et al. [30] reported in kale. The difference among studies are probably due to differences in crops and cultivars analyzed, growing conditions, analytical procedures, and different physiological stages of sample.

Carotenoids have been studied for their potential health benefits, including against cancers, cardiovascular diseases, and skin diseases [10]. Additionally, carotenoids have shown beneficial impact on eye health, as some carotenoids such as β-carotene possess provitamin A activity [31]. In order to estimate nutritional value related to β-carotene of the mustard cultivars used in this study, we calculated %RDA value of vitamin A based on 100 g of fresh mustard leaves using water content reported by the United State Department of Agriculture (USDA) nutrient database [32] and retinol activity equivalents [33]. ‘Jeok’ offered the highest level of %RDA of vitamin A, providing 64.1–66.5% and 82.4–85.5% of the RDA values for male and female (>14 years), respectively, depending on maturity (Supplementary Table S2). In contrast, ‘Amara’ (22.0–28.3%) and ‘Suehling’ (24.9–32.0%) were the lowest in %RDA of vitamin A at baby leaf and mature stages, respectively. This result indicates that mustard leaves, depending on cultivar, can be an excellent source of β-carotene and thus beneficial for eye health due to their provitamin A activity. However, it has been reported that bioavailability and vitamin A equivalency of β-carotene greatly varies depending on food/diet-related conditions as well as the characteristics of human population [34]. Haskell [34] reported that the absorption of β-carotene from plant resources can be as low as 5% and the vitamin A equivalency varies depending on the food matrix. Although there is a relatively complex food matrix of vegetables and potentially low absorption of β-carotene from such sources [34], mustard leaves can still be a good source of β-carotene and vitamin A due to their high concentration. Although not directly related to vitamin A activity, other carotenoids such as lutein have also been reported for their potential health benefits [10].

### 2.4. Total Anthocyanin Concentrations (TAC)

Anthocyanins are the major pigment responsible for red-purple pigmentation of the leaves of red mustard cultivars. Total monomeric anthocyanin content ranged from 1.9 μg/g DW in immature ‘Amara’ to 1985.4 μg/g DW in mature ‘Garnet’ (Table 3), indicating a high level of anthocyanin in red cultivars and very low concentration in green cultivars. It was previously reported that a transcription factor *TT8* probably plays a key role in purple *B. juncea* plant, triggering anthocyanin biosynthesis, compared to a green cultivar [5,35]. To our knowledge, information on phytonutrient profile of mustard is relatively limited, but a number of acylated cyanidin-based anthocyanins have been identified in red mustard leaves [36]. There was a significant correlation of the total anthocyanin concentrations between baby leaf and mature stages (*r* = 0.8647, *p* < 0.001; Table 1). However, not all cultivars showed the same trend in anthocyanin change with the growth of mustards. For instance, the total anthocyanin content in ‘Garnet’ numerically increased, although non-significant, from 1477.8 to 1985.4 μg/g DW from baby leaf to mature stage, but baby leaf ‘Ruby Streaks’ (1261.2 μg/g DW) had a significantly higher TAC than mature ‘Ruby Streaks’ (449.3 μg/g DW; *p* = 0.024). This result indicates that even within a crop, there can be a different regulatory mechanism for biosynthesis and accumulation of anthocyanins among mustard cultivars. To date, the health benefits of mustard have not been well investigated, but anthocyanin-rich lettuce, where cyanidin-based anthocyanins are predominant similar to mustard, has shown anti-diabetic activity by reducing hyperglycemia and improving insulin sensitivity in male C57Bl/6J fed high-fat diet [37]. Similarly, male Wistar rats fed a diet containing 20% red oak leaf lettuce had lower ratio of low density lipoprotein cholesterol to high density cholesterol in plasma, liver cholesterol level, and cholesterol absorption, indicating a potential benefit against the risk of cardiovascular diseases [38]. Among investigated cultivars, ‘Garnet’ had the highest concentration of anthocyanins in both the baby leaf (1477.8 μg/g DW) and mature mustard (1985.4 μg/g DW), indicating that this cultivar may provide greater anthocyanin-related health benefits compared to the other cultivars regardless of maturity.

### 2.5. Total Phenolics and Antioxidant Capacity

Total phenolic concentration (TPC) varied among cultivars in both baby leaf and mature mustard. TPC ranged from 4.14 mg/g DW in ‘Pacific Gold’ to 7.48 mg/g DW in ‘Red Splendor’ in baby leaf stage, while ranging from 2.60 mg/g DW in ‘Jeok’ to 4.14 mg/g DW in ‘Garnet’ in mature leaves (Table 3). We found that TPC decreased or did not change from baby leaf to mature stage, depending on cultivar. Phenolic content in mustard has been reported in few studies. Fang et al. [39] reported 7.95 mg/g DW of TPC, and Harbaum et al. [40] reported a similar TPC, from 2.72 to 8.26 m/g DW, depending on cultivar and growth condition. To our knowledge, our study is the first report on TPC in various mustard cultivars at different physiological stages.

The DPPH assay was employed to estimate total antioxidant capacity of mustard leaves, and ‘Ruby Streaks’ was found to be the highest in antioxidant capacity at both physiological stages. Other cultivars ‘Amara’ and ‘Garnet’ were also high in antioxidant capacity in baby leaf and mature plants, respectively. In contrast, low antioxidant capacity was found in ‘Dol San’ and ‘Jeok’, despite physiological stage. Antioxidant assays including DPPH method may reflect phenolic content in the sample. In fact, we found a significant correlation between antioxidant capacity and total anthocyanin (*r* = 0.4986, *p* = 0.0182, *n* = 22) or TPC (*r* = 0.4480, *p* = 0.0365, *n* = 22, Table 1). However, this result was primarily due to mature plants, as there was no significant correlation found between antioxidant capacity and total anthocyanin or TPC for baby leaf stage. In mature mustard, antioxidant capacity was significantly correlated with both anthocyanin content (*r* = 0.6608, *p* = 0.0268, *n* = 11) and TPC (*r* = 0.7609, *p* = 0.0065, *n* = 11). This result indicates that phenolic compounds including anthocyanin were the primary factor contributing to antioxidant capacity analyzed by DPPH assay in mature mustard leaves. However, there may be other factors influencing antioxidant capacity of mustard at baby leaf stage, suggesting that further studies are needed to better understand antioxidant activity of mustard at younger stage.

## 3. Materials and Methods 

### 3.1. Mustard Cultivation

Seeds of eight *Brassica juncea* cultivars and one *B. carinata* (‘Amara’) were purchased from Johnny’s Selected Seeds (Winslow, ME, USA). Seeds of two *B. juncea* cultivars, ‘Jeok’ and ‘Dol San’, were purchased from Asia Seed (Seoul, Korea). Seeds were germinated in a 36-cell tray filled with Sunshine LC1 (Sun Gro Horticulture, Vancouver, BC, Canada). Mustard seedlings were grown in a greenhouse (West Virginia University, Morgantown, WV, USA), where the temperature was set at 24/20 °C (day/night), under natural irradiance. Twenty of baby leaf stage-plants were harvested for a biological replication (total *N* = 3) 19 days after sowing. Then, rest of plants were transplanted to a 24-cm pot filled with Sunshine LC1 (Sun Gro Horticulture, Vancouver, BC, Canada) and grown for additional 16 days. Three mature plants were harvested for a biological replication (total *N* = 3). Plants were irrigated as needed and 20% Hoagland solution was provided once a week after transplanting. Immediately after each harvest, mustard leaf samples were weighed for whole aerial part of fresh weight and frozen in liquid nitrogen, lyophilized, ground to a fine powder using a coffee grinder, and stored at −20 °C until analyses.

### 3.2. Quantitation of Glucosinolate

Glucosinolates were analyzed following the methods of Ku et al. [15] and Kliebenstein et al. [41] with minor modifications. Freeze-dried mustard leaf powder from each of the 11 cultivars (50 mg) was weighed into a 0.5 mL screwcap vial (USA Scientific, Ocala, FL, USA) and mixed with 2 mL of 70% methanol. After heating at 95 °C for 10 min in a heating block, tubes were cooled on ice for 5 min, followed by the addition of internal standard (0.907 mM glucosinalbin, isolated from *Sinapis alba*). Tubes were vortexed and centrifuged at 12,000× *g* for 10 min at room temperature and the supernatants were collected. The pellets were extracted again with 0.5 mL of 70% methanol. 

The pooled extract was mixed with 0.15 mL of a mixture of 1 M lead acetate and 1 M barium acetate (1:1, *v*/*v*) and vortexed for protein precipitation. After centrifuging at 12,000× *g* for 1 min, contents of each tube were poured into a drained poly-prep column containing DEAE Sephadex A-25 resin (0.2 mL, 1:1 (*v*/*v*) in water) (GE Healthcare, Piscataway, NJ, USA) pre-charged with 1 M NaOH and 1 M pyridine acetate. After the sample solution passed through the resin, 3 mL of 0.02 M pyridine acetate was added, followed by the addition of 3 mL of deionized distilled water. Once deionized distilled water passed through the resin, samples were incubated with 0.5 mL of sulfatase solution overnight at room temperature. Then, glucosinolates were eluted with 3 mL deionized distilled water. Tubes were vortexed and the eluent was filtered through a 0.22 μm Nylon syringe filter into an HPLC vial.

Filtered sample of 1 μL was injected into a Nexera-i, LC 2040C ultra-high performance liquid chromatography (UHPLC) (Shimadzu, Kyoto, Japan) equipped with photo diode array detector. A Reversed-phase C18 column (100 mm × 2.1 mm i.d., 1.8 μm, 100 Å; AkzoNobel, Bohus, Sweden) was used to analyze glucosinolates. Deionized distilled water (mobile phase A) and 100% acetonitrile (mobile phase B) were used with the following gradient condition: 0 min 2.5% B, 1 min 2.5% B, 2 min 4% B, 3.5 min 18% B, 7 min 25% B, 7.25 min 80% B, 8 min 80% B, and 8.1 min 2.5% B with a flow rate of 0.3 mL/min. The oven temperature was 40 °C. Glucosinolates were detected at 229 nm and identified by comparing with previous study [42] and then quantified using internal standard and relative response factor [43].

### 3.3. Hydrolysis Products and Nitrile FORMATION (%)

Glucosinolate hydrolysis products were analyzed using a gas chromatography-mass spectrometry (GC-MS) following the method of Kim et al. [44]. Freeze-dried mustard powder (50 mg) was mixed with 1 mL of deionized distilled water. After vortexing, samples were centrifuged at 12,000× *g* for 2 min, then 0.5 mL of extract was transferred to a Teflon tube (Savillex Corporation, Eden Prairie, MN, USA), followed by addition of 0.5 mL of dichloromethane. Samples were incubated for 4 h to hydrolyze glucosinolates by an endogenous enzyme myrosinase. Then, tubes were centrifuged at 12,000 *g* for 2 min and the lower organic layer was collected. Incubation time was determined from a preliminary study from various incubation times from 0.5, 4, 8, 16, and 24 h (Supplementary Figure S3).

A GC (Trace 1310 GC, Thermo Fisher Scientific, Waltham, MA, USA) coupled to a MS detector system (ISQ QD, Thermo Fisher Scientific, Waltham, MA, USA) and an autosampler (Triplus RSH, Thermo Fisher Scientific, Waltham, MA, USA) was used for the analysis of glucosinolate hydrolysis products. A capillary column (DB-5MS, Agilent Technologies, Santa Clara, CA, USA; 30 m × 0.25 mm × 0.25 μm capillary column) was used. After an initial temperature held at 35 °C for 1 min, the oven temperature was increased to 310 °C at a rate of 40 °C/min and held for 5 min. Inlet and ion source temperatures were set at 270 °C and 300 °C, respectively. Mass spectra were obtained by electron ionization mode with the scan range of 40–450 *m*/*z*. The flow rate of the helium carrier gas was set at 1.2 mL/min. AITC and CETP were quantified using a standard curve of AITC. The nitrile formation (%) was calculated as a relative ratio of AITC and CEPT to the total hydrolysis products to estimate ESP activity.

### 3.4. Carotenoid Analysis

Carotenoids were extracted following the method of Maurer et al. [45] with slight modifications. Freeze-dried powder (0.1 g) of each sample was extracted with 8.5 mL of acetone/methanol (2:1, *v*/*v*, containing 0.5% BHT). Then, 3 mL of hexane containing 0.5% BHT and internal standard (β-apo-8′-carotenal) were added, followed by sonication in ice water for 15 min. After vigorous shaking, 8 mL of cold sodium chloride (1 M) was added, followed by centrifugation at 1800 rpm for 10 min for phase separation. Upper hexane layer was filtered through a 0.2 μm PTFE syringe filter to an HPLC amber vial.

Carotenoids were analyzed using a Nexera-i, LC UHPLC (Shimadzu, Kyoto, Japan) equipped with photo diode array detector. Acclaim C30 column (Thermo Scientific, Waltham, MA, USA) was used for the analysis. Deionized distilled water, acetonitrile, methanol/ethyl acetate (1:1, *v*/*v*), and 200 mM acetic acid in water were mixed in water for mobile phase A, B, C, and D, respectively, with the following gradient: 0–1 min, 75% B, 10% C, 0.5% D; 1.5 min, 70% B, 20% C, 0.5% D; 8 min, 70% B, 29.5% C, 0.5% D; 9.5 min, 50% B, 49.5% C, 0.5% D; 15 min, 50% B, 49.5% C, 0.5% D; 15.1 min, 75% B, 10% C, 0.5% D. The flow rate was 1.5 mL/min. The oven temperature was set at 40 °C and each sample was run for 19 min. The injection volume was 5 μL and carotenoids were detected at 450 nm. Each carotenoid was identified using an authentic standard and quantified using relative response factor.

### 3.5. Total Anthocyanin Analysis

Total anthocyanin content was analyzed following the method of Kim et al. [46], with slight modifications. Freeze-dried powder (50 mg) was extracted twice in 1 mL of acidified methanol (60:37:3, methanol: deionized distilled water: acetic acid, *v*/*v*/*v*). The pH differential method was used for total anthocyanin analysis. Each sample (300 μL) was mixed with either 1200 μL of 0.025 M potassium chloride (pH 1.0) or 0.4 M sodium acetate (pH 4.5). After 60 min of incubation at room temperature in the dark, samples were read at 510 and 700 nm using an Epoch 2 plate reader (Biotek Instruments Inc., Winooski, VT, USA). Total anthocyanin concentration was quantified as cyanidin 3-glucoside equivalent.

### 3.6. Total Phenolic Content and Antioxidant Capacity

Total phenolic content and antioxidant capacity were analyzed following the method by Ku et al. [47] with minor modification. Freeze-dried mustard leaves (75 mg) were extracted in 6 mL of 70% methanol. The extracts were used for the total phenolic content and antioxidant capacity analysis. Total phenolic content was analyzed using Folin-Ciocalteu assay. Each sample (20 μL) was mixed with (100 μL) of Folin-Ciocalteu reagent (0.2 N), followed by 3 min of incubation at room temperature. Then, 50 μL of sodium carbonate (2.0%) was added. After 60 min of incubation in the dark at room temperature, absorbance was obtained at 735 nm. Total phenolic concentration was determined based on a standard curve of gallic acid. All samples were analyzed in triplicate.

The DPPH assay was employed to measure antioxidant capacity of the mustard samples. Reaction mixtures containing test samples (10 μL) and 190 μL of a 200 μM DPPH in ethanol were incubated at room temperature for 30 min in 96-well plates. The absorbance of the DPPH free radical was measured at 515 nm using an Epoch 2 plate reader (Biotek Instruments Inc., Winooski, VT, USA). Results were expressed in percentage of scavenging activity compared to control (extraction solvent). Samples were assayed in triplicate.

### 3.7. Statistical Analysis

Each replication consisted of 20 plants for baby leaf stage except for one replication in ‘Amara’ (*N* = 11) and 3 plants for mature stage. One-way ANOVA and Tukey’s honest significant difference (HSD) test were performed using JMP 12 (SAS, Cary, NC, USA) to assess the effect of cultivar on phytonutrients and antioxidant capacity. Pearson’s correlation was conducted on all pairs of data based on the mean values of each cultivar from two physiological stages.

## 4. Conclusions

In this study, we analyzed glucosinolates and their hydrolysis products, carotenoids, and total anthocyanin and phenolic contents in 11 mustard cultivars at two different physiological stages. The glucosinolate profile was consistent in all cultivars with sinigrin being the predominant glucosinolate. However, their concentration and their change from baby leaf to mature stage were cultivar-dependent. AITC and CETP were the major hydrolysis products, and their relative ratio differed among cultivars, indicating potentially different ESP activity and bioactivity among cultivars. We found 4 carotenoids, β-carotene, lutein, violaxanthin, and neoxanthin, with their concentrations depending on cultivar and the physiological stage. Our results indicate that mustard leaves can be an excellent source of vitamin A, based on the %RDA of vitamin A calculated using β-carotene content and retinol activity equivalents. Mustard leaves were also a source of phenolic compounds, including anthocyanins. Their concentration differed between the baby leaf and mature stages, but depending on the cultivar. Antioxidant capacity analyzed by DPPH assay was positively correlated with total anthocyanin and phenolic contents in mature mustard leaves. To our knowledge, this is the first study reporting various groups of phytochemicals in different mustard cultivars. Moreover, how these phytochemicals change from baby leaf to mature plants has not been well reported for mustard. Therefore, our results provide baselines of the phytochemical profiles of various mustard cultivars at different physiological stages and will assist growers and consumers in selecting mustard cultivars with greater health benefits. Additionally, our result can also assist further studies on phytonutrient composition and its change with the physiological development of other vegetables to develop vegetables with greater health benefits.

## Figures and Tables

**Table 1:** Selected significant correlation coefficients for phytochemical concentration and antioxidant capacity of 11 mustard cultivars.

Variable	by Variable	Correlation Coefficient
Glucobrassicin (mature)	Glucobrassicin (baby leaf)	0.8072 **^,a^
Neoglucobrassicin (mature)	Neoglucobrassicin (baby leaf)	0.7275 **
Total indole glulcosinolates (mature)	Total indole glulcosinolates (baby leaf)	0.7783 **
Nitrile formation from sinigrin (mature)	Nitrile formation from sinigrin (baby leaf)	0.8785 **
Nitrile formation from glucobrassicin (mature)	Nitrile formation from glucobrassicin (mature)	0.9164 ***
Total phenolics (mature)	Total phenolics (baby leaf)	0.7495 **
Anthocyanins (mature)	Anthocyanins (baby leaf)	0.8647 ***
Total phenolics (mature)	Anthocyanins (mature)	0.8262 **
Total phenolics (mature)	Antioxidant capacity—DPPH (mature)	0.7609 **
Antioxidant capacity—DPPH (mature)	Anthocyanins (mature)	0.6608 *

^a^ Asterisks *, **, and *** indicate the significant correlation at *p* ≤ 0.05, 0.01, and 0.001, respectively.

**Table 2:** Carotenoid concentrations of mustard leaves at baby leaf (upper values in the cells) and mature (bottom values in the cells) stages.

Cultivar Name	β-Carotene	Violaxanthin	Neoxanthin	Lutein
	μmole/g DW			
Amara	476 ± 26 c ^a^	830 ± 32 bcd	213 ± 12 ab	1445 ± 88 a
	580 ± 108 c	1153 ± 185 abc	294 ± 51 a	1485 ± 289 ab
Dol San	931 ± 258 abc	838 ± 52 bcd	158 ± 8 bc	1261 ± 71 a
	1113 ± 163 ab	1093 ± 104 abc	299 ± 17 a	2039 ± 11 a
Garnet	912 ± 67 bc	654 ± 12 d	142 ± 3 c	1335 ± 55 a
	804 ± 50 bc	1056 ± 14 abc	273 ± 13 a	1637 ± 31 ab
Golden Frill	710 ± 58 bc	771 ± 23 cd	154 ± 3 bc	1388 ± 61 a
	664 ± 207 bc	957 ± 120 bc	268 ± 20 a	1582 ± 96 ab
Jeok	1438 ± 21 a	647 ± 30 d	192 ± 5 abc	1260 ± 40 a
	1386 ± 98 a	767 ± 80 c	272 ± 31 a	1901 ± 218 ab
Pacific Gold	696 ± 84 bc	1027 ± 10 ab	227 ± 8 a	1533 ± 23 a
	704 ± 20 bc	1461 ± 31 a	293 ± 9 a	1649 ± 48 ab
Red Giant	717 ± 94 bc	1114 ± 88 a	228 ± 30 a	1595 ± 118 a
	644 ± 21 bc	1205 ± 53 abc	272 ± 16 a	1458 ± 52 ab
Red Splendor	666 ± 24 bc	866 ± 24 bc	189 ± 6 abc	1375 ± 57 a
	637 ± 23 bc	1278 ± 115 ab	303 ± 28 a	1627 ± 193 ab
Ruby Streaks	675 ± 147 bc	879 ± 42 bc	182 ± 12 a	1239 ± 116 a
	863 ± 93 bc	992 ± 34 bc	267 ± 7 a	1655 ± 36 ab
Southern Giant Curled	1114 ± 44 ab	815 ± 39 cd	178 ± 11 abc	1557 ± 79 a
	573 ± 37 c	988 ± 38 bc	256 ± 9 a	1482 ± 62 ab
Suehling	782 ± 60 bc	869 ± 15 bc	185 ± 4 abc	1407 ± 73 a
	539 ± 12 c	1071 ± 68 abc	261 ± 26 a	1339 ± 84 b

^a^ Data are presented as mean ± standard error (total *n* = 3). Different letters next to number indicate that mean values were significantly different within cultivar at each physiological stage by Tukey’s HSD at *p* ≤ 0.05.

**Table 3:** Total Anthocyanin Content (TAC), Total Phenolics, and DPPH (antioxidant) concentrations in the mustard at baby leaf (upper values in the cells) and mature (bottom values in the cells) stages.

Cultivar Name	TAC	Total Phenolics	Antioxidant Capacity
	μg/g DW	mg/g DW	%
Amara	1.9 ± 1.3 c ^a^	4.15 ± 0.11 c	40.6 ± 1.9 a
	7.8 ± 2.2 d	2.83 ± 0.12 cde	29.2 ± 1.3 bc
Dol San	9.3 ± 2.9 c	4.63 ± 0.19 bc	18.8 ± 0.8 d
	4.8 ± 2.0 d	2.73 ± 0.06 e	23.9 ± 0.2 c
Garnet	1477.8 ± 98.7 a	5.71 ± 0.26 b	30.2 ± 2.1 bc
	1985.4 ± 127.5 a	4.14 ± 0.13 a	37.9 ± 1.2 a
Golden Frill	5.6 ± 1.9 c	4.18 ± 0.13 c	22.0 ± 1.2 cd
	16.0 ± 4.3 d	3.11 ± 0.08 cde	26.6 ± 1.7 bc
Jeok	40.4 ± 4.5 c	4.68 ± 0.19 bc	21.7 ± 2.0 cd
	39.3 ± 9.2 d	2.60 ± 0.15 e	20.8 ± 1.3 c
Pacific Gold	13.0 ± 2.0 c	4.14 ± 0.10 c	31.0 ± 1.6 bc
	4.18 ± 0.7 d	2.74 ± 0.11 de	28.2 ± 1.9 bc
Red Giant	300.1 ± 8.7 c	5.36 ± 0.19 bc	33.9 ± 2.4 ab
	335.5 ± 90.0 c	3.00 ± 0.09 cde	24.9 ± 1.5 c
Red Splendor	733.8 ± 51.5 b	7.48 ± 0.71 a	37.9 ± 1.0 ab
	673.9 ± 35.3 b	3.85 ± 0.19 ab	33.7 ± 3.7 ab
Ruby Streaks	1261.2 ± 214.6 a	5.36 ± 0.19 bc	40.7 ± 1.4 a
	449.3 ± 81.1 bc	3.40 ± 0.25 bcd	41.4 ± 1.8 a
Southern Giant Curled	10.4 ± 3.2 c	5.36 ± 0.10 bc	35.2 ± 2.7 ab
	21.2 ± 13.7 d	3.43 ± 0.03 bc	26.4 ± 0.3 bc
Suehling	12.4 ± 7.2 c	4.65 ± 0.03 bc	32.7 ± 2.3 ab
	10.8 ± 2.6 d	2.68 ± 0.05 e	21.7 ± 0.9 c

^a^ Data are presented as mean ± standard error (total *n* = 3). Different letters next to number indicate that mean values were significantly different within cultivar at each physiological stage by Tukey’s HSD at *p* ≤ 0.05.

## Supplementary Materials

Supplementary materials are available online.
