# Supplementary material for: Health-Promoting Phytochemicals from 11 Mustard Cultivars at Baby Leaf and Mature Stages
Source: Molecules. 2017 Oct 17;22(10):1749. doi: 10.3390/molecules22101749 (PMC6151555; doi:10.3390/molecules22101749)
Supplement: Supplementary file 1 [file molecules-22-01749-s001.pdf]

Health-promoting phytochemicals from 11 mustard cultivars at baby leaf and mature stages

Marissa D. Frazie [1#], Moo Jung Kim [2#] and Kang-Mo Ku [2,*]

[1] Division of Animal and Nutritional Sciences, West Virginia University, Morgantown, WV 26506, USA
[2] Division of Plant and Soil Sciences, West Virginia University, Morgantown, WV 26506, USA
# Equally contributed
* Correspondence: kangmo.ku@mail.wvu.edu; Tel.: +1 304-293-2549

Table S1. Mean weight (g DW/plant) of 11 mustard cultivars at baby leaf and mature stage.

**Cultivar**	**Baby leaf**	**Mature**
Amara	0.30[z]	6.01
Dol San	0.36	7.38
Garnet	0.26	7.27
Golden Frill	0.34	4.77
Jeok	0.29	6.32
Pacific Gold	0.36	6.04
Red Giant	0.33	6.38
Red Splendor	0.25	7.07
Ruby Streaks	0.21	4.73
Southern Giant Curled	0.25	4.35
Suehling	0.27	7.41

[z]Dry weight per plant was calculated using fresh weight measured at harvest and moisture content (90.7%) from the USDA database [1]. Means were separated within cultivar at each physiological stage by Tukey's HSD at $p \leq 0.05$.

Table S2. %RDA values for vitamin A for male and female (> 14 years) from baby leaf and mature mustard cultivars based on β-carotene concentration.

Cultivar Name	%RDA	
	For male	For female
Amara	22.0	28.3
	26.8	34.5
Dol San	43.1	55.4
	51.5	66.2
Garnet	42.1	54.2
	37.2	47.8
Golden Frill	32.8	42.2
	30.7	39.5
Jeok	66.5	85.5
	64.1	82.4
Pacific Gold	32.2	41.4
	32.6	41.9
Red Giant	33.2	42.6
	29.8	38.3
Red Splendor	30.8	39.6
	29.5	37.9
Ruby Streaks	31.2	40.1
	39.9	51.3
Southern Giant Curled	51.5	66.2
	26.5	34.0
Suehling	36.2	46.5
	24.9	32.0

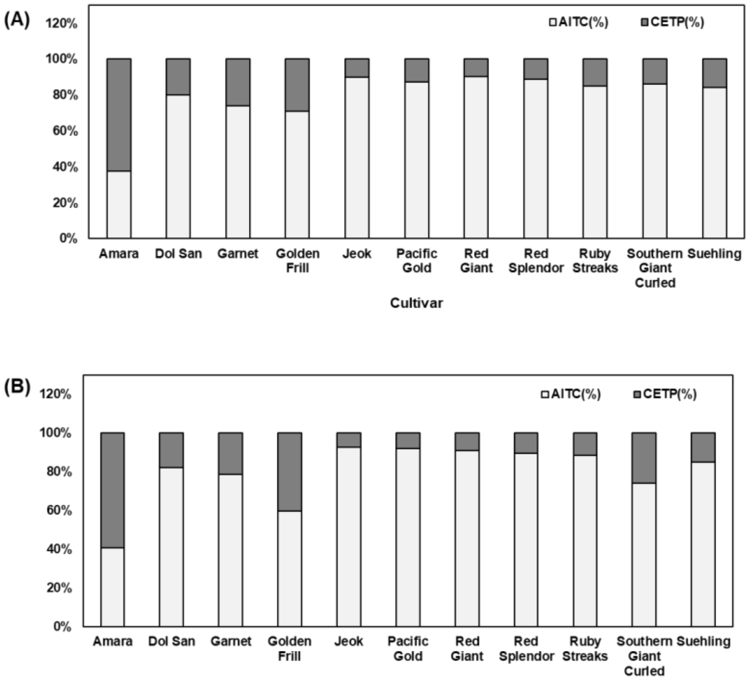

Figure S1. Relative ratio of AITC and CETP of 11 mustard leaves at (A) baby leaf and (B) mature stages. AITC, allyl isothiocyanate; CETP, 1-cyano-2,3-epithiopropane.

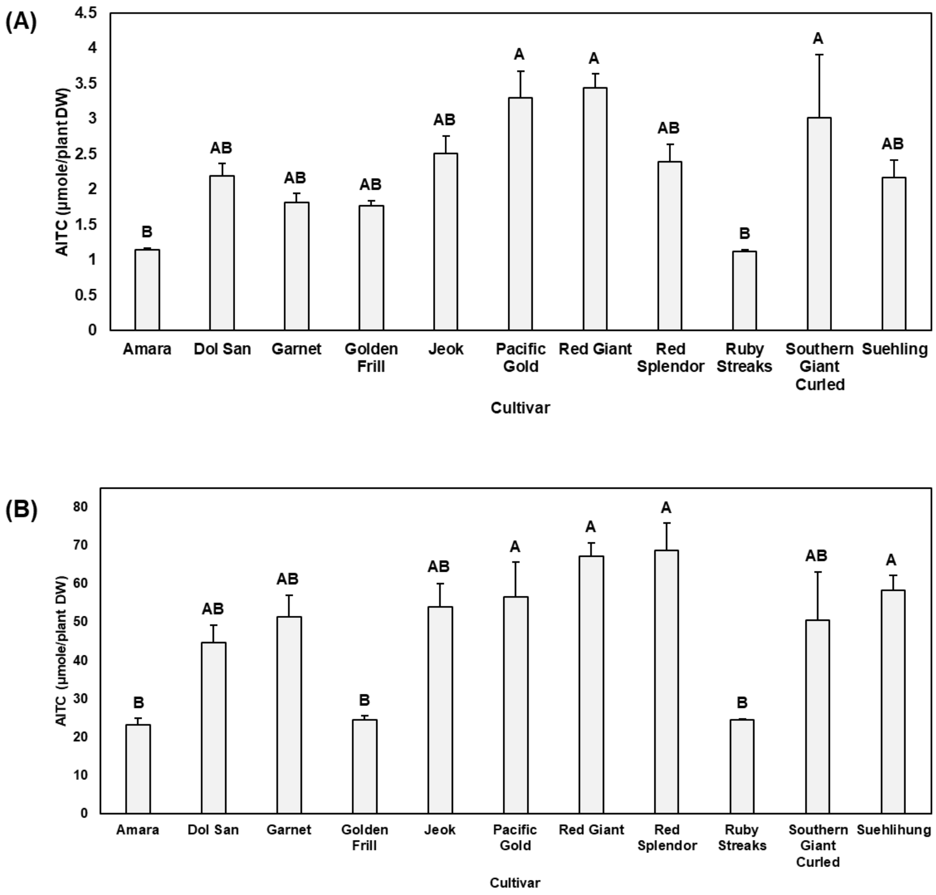

Figure S2. AITC produced per plant at baby leaf and mature stages. Vales were calculated using AITC concentration shown in Figure 2 and dry weight per plant shown in Table S1. Means were separated within cultivar at each physiological stage by Tukey's HSD at $p \leq 0.05$.

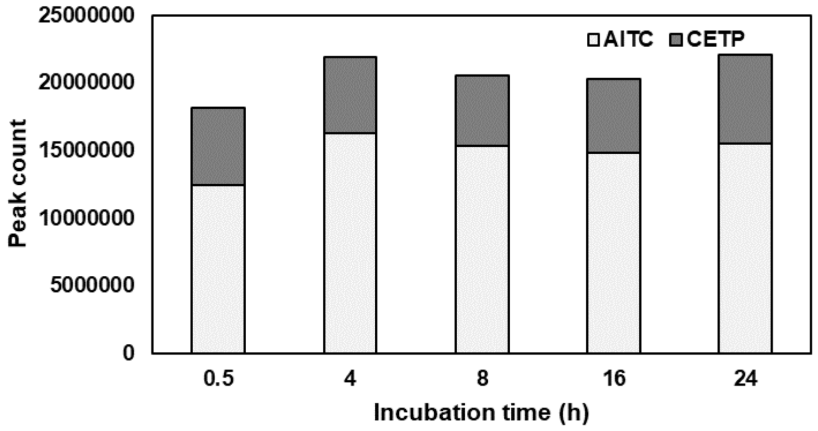

Figure S3. AITC and CETP produced after different incubation time. Data are the average of 3 replications. AITC with the incubation time of 4, 8, and 24 h was significantly higher than 0.5 h of incubation, but CETP and the total peak count of both compounds did not significantly differ among different incubation times by Tukey's HSD at $p \leq 0.05$.

Reference

1. USDA. National Nutrient Database for Standard Reference Release 28. http://ndb.nal.usda.gov/ndb/ (Mar. 20 2017),
